# Involvement of Integrin-Activating Peptides Derived from Tenascin-C in Cancer Aggression and New Anticancer Strategy Using the Fibronectin-Derived Integrin-Inactivating Peptide

**DOI:** 10.3390/molecules25143239

**Published:** 2020-07-16

**Authors:** Motomichi Fujita, Manabu Sasada, Takuya Iyoda, Fumio Fukai

**Affiliations:** 1 Department of Molecular Patho-Physiology, Faculty of Pharmaceutical Sciences, Tokyo University of Science, 2641 Yamazaki, Noda, Chiba 278-8510, Japan; motomichi.f@gmail.com (M.F.); ssdy1321.tus@gmail.com (M.S.); 2 Clinical Research Center in Hiroshima, Hiroshima University Hospital, 1-2-3 Kasumi, Minami-Ku, Hiroshima 734-8551, Japan; 3 Department of Pharmacy, Faculty of Pharmaceutical Sciences, Sanyo-Onoda City University, 1-1-1 Daigaku-Doori, Sanyo-Onoda, Yamaguchi 756-0884, Japan

**Keywords:** extracellular matrix, matricellular protein, cell adhesion, β1-integrin, α5-integrin tenascin-C, fibronectin, glioma, glioblastoma, colitis-associated colorectal cancer

## Abstract

Matricellular proteins, which exist in association with the extracellular matrix (ECM) and ECM protein molecules, harbor functional sites within their molecular structures. These functional sites are released through proteolytic cleavage by inflammatory proteinases, such as matrix metalloproteinases (MMPs) and a disintegrin and metalloproteinase with thrombospondin motifs (ADAMTS), and the peptides containing these functional sites have unique biological activities that are often not detected in the parent molecules. We previously showed that tenascin-C (TNC) and plasma fibronectin (pFN), examples of matricellular proteins, have cryptic bioactive sites that have opposite effects on cell adhesion to the ECM. A peptide containing the bioactive site of TNC, termed TNIIIA2, which is highly released at sites of inflammation and in the tumor microenvironment (TME), has the ability to potently and persistently activate β1-integrins. In the opposite manner, the peptide FNIII14 containing the bioactive site of pFN has the ability to inactivate β1-integrins. This review highlights that peptide TNIIIA2 can act as a procancer factor and peptide FNIII14 can act as an anticancer agent, based on the regulation on β1-integrin activation. Notably, the detrimental effects of TNIIIA2 can be inhibited by FNIII14. These findings open the possibility for new therapeutic strategies based on the inactivation of β1-integrin by FNIII14.

## 1. Introduction

Extracellular matrix (ECM) molecules, such as fibronectin (FN), collagen, and laminin, serve as the molecular and structural scaffold for cell adhesion and for the maintenance of tissue architecture and tissue polarity. Unlike structural ECM molecules, secreted non-structural ECM components called matricellular proteins serve to modulate cell–cell and cell–matrix interactions. Families of matricellular proteins include tenascins, osteopontin, secreted protein acidic and rich in cysteine (SPARC) family members, and thrombospondins, and these are characterized by high expression levels during development and in response to injury [1]. They bind to the ECM to modulate a variety of biological signals for cell regulation, including survival, death, proliferation, migration, differentiation, and gene expression [2]. These cell pathways modulated by ECM molecules are mainly regulated by cell adhesion receptors known as integrins.

Integrins are heterodimeric membrane-spanning receptors of α and β subunits that transmit information from the ECM to the cell through the activation of cell-signaling pathways. In mammals, 18 different integrin α subunits and 8 different integrin β subunits have been identified to date, and they are able to combine noncovalently to form 24 unique heterodimers [3]. Combinations of α and β subunits of integrins determine the binding specificity of the ligand [3]. In contrast to transmembrane receptors for humoral factors, such as cytokines and growth factors, integrins have the unique ability to alter the binding affinity of their ligands. Integrins exist largely in two different structural states: an inactive conformation without ligand-binding ability (bent form) and an active one with high affinity for ligand binding (extended form) [4]. The conversion of integrins between these states is reversible [5]. The conversion from the inactive to the active conformation is mainly triggered by a unique bidirectional signaling pathway (referred to as “inside-out” and “outside-in” signaling) [6]. Intracellular proteins in cells stimulated by humoral factors, such as chemokines or cytokines, are activated, and the cytoplasmic domain of the integrin β subunit leads to the binding of integrin-associated proteins such as talin and kindlins, and the formation of focal adhesions, resulting in alteration and retention within the integrin conformation to an activated state with high ligand binding affinity (“inside-out” signaling). Moreover, upon binding to extracellular ligands, integrin activation occurs and transduces a signal to cytoplasm, resulting in the formation of focal adhesions (“outside-in” signaling). In addition, the conformational shift of integrins from the inactive to active state also occurs via direct or indirect association with other cell surface proteins, such as syndecan or tetraspanin. These cells result in the acquisition of adhesive properties and, consequently, the expression of distinctive functions [6].

In addition to the unique properties in the conformational regulation of integrins, integrin-mediated cell signaling via ECM molecules is affected by functional bioactive sites within the ECM protein molecules or matricellular proteins [7]. Matricellular proteins and ECM protein molecules harbor functional sites within their molecular structures [7,8]. Some of these functional sites are exposed on the ECM surface, such as the Arg–Gly–Asp (RGD)-motif in fibronectin and vitronectin [9], while others are instead usually embedded within ECM protein molecules [8]. These hidden functional sites, referred to as matricryptic sites, are revealed through proteolytic cleavage by inflammatory proteinases and/or the structural unfolding of these molecules based on cell adhesion and intermolecular interactions in accordance with the temporal and spatial alteration of the microenvironment [7,8]. These cryptic functional sites have unique biological activities that are often not detected in the parent molecules [8]. Most fragments/peptides containing these cryptic functional sites express their biological activities through the direct binding to integrins [10,11]. Previous reports showed that laminin also harbors several cryptic functional sites, and some of them have the ability to regulate the adhesion, proliferation, migration, and metastasis in cancer cells under in vitro and in vivo settings [12,13,14]. Furthermore, based on the biological function of these fragments/peptides, a number of previous studies have suggested that synthetic peptides and peptidomimetics as specific integrin-targeted agents, such as cilengitide and ATN-161 (please see “Section 4”), are considered attractive therapeutic applications [11,15]. Among them, it has been shown that peptides related to the RGD motif, which is the integrin-binding sequence commonly found in several ECM protein molecules, such as FN, vitronectin, laminin, and osteopontin, can be developed as anticancer therapeutics. While several peptidic agents have shown anticancer activity in preclinical models, these agents have failed to show substantial benefits in clinical trials [16]. These peptides derived from the integrin recognition sequence have been created in an attempt to develop integrin signal blockers based on the competitive inhibition of cell–ECM molecule interactions. However, they cannot induce the conformational change of integrins under pharmacologically effective concentrations. Given that active or inactive states of integrins respectively determine the specific signaling pathways, the action of the integrin competitive antagonists seems inherently limited.

To date, a number of studies have reported the presence of bioactive fragments and matricryptic sites within tenascin-C (TNC) and FN (Table 1). Among them, we previously found that TNC and plasma fibronectin (pFN)—typical matricellular proteins—have cryptic bioactive sites in their molecules that produce opposite effects on cell adhesion to the ECM. A peptide containing the bioactive site of TNC, termed TNIIIA2, which is highly expressed in inflammatory regions and the tumor microenvironment (TME), has the ability to potently and persistently activate β1-integrins. Based on these activities, TNIIIA2-containing TNC fragments/peptides are involved in the acquisition of aggressiveness in cancer progression. In the opposite manner, the peptide containing the bioactive site of pFN, termed FNIII14, has the ability to inactivate β1-integrins. Of particular note, FNIII14 can inhibit the acquisition of malignant properties in response to TNIIIA2-induced β1-integrin activation. It should be emphasized that FNIII14 blocks integrin signaling by a mechanism entirely distinct from that of integrin competitive antagonists [17]. The inactivation of β1-integrin by FNIII14 may result in antitumor effects not achievable with competitive inhibitors of integrin–ECM binding.

## 2. Tenascin-C-Derived Peptide, TNIIIA2

TNC is a multifunctional glycoprotein that belongs to a family of matricellular proteins [49]. It is highly expressed during embryonic development, but its expression levels rapidly decrease and are limited in normal adult tissues [49]. TNC is strongly re-expressed in particular environments such as inflammatory regions [50], tissue remodeling [51,52,53], and the TME [54], indicating a close association with pathogenesis. A high expression of TNC has been observed in various cancers including pancreatic cancer [55], gastric cancer [56], colorectal cancer [57], esophageal adenocarcinoma [58], lung cancer [59], breast cancer [60,61], hepatocellular carcinoma [62], cholangiocarcinoma [63], prostate cancer [64], oral tongue squamous cell carcinoma [65], and glioma/glioblastoma [54,66]. High TNC levels are correlated with poor prognosis in patients with various types of cancer, and its expression is therefore considered a poor prognostic factor. Besides cancer cells, other cells in the TME including fibroblasts, endothelial cells, and macrophages express TNC, thus contributing to cancer aggression [67,68].

The features of the excessive survival/proliferation and disseminative migration in cancer cells is considered the result of malignant acquirement in the context of the TME. Therefore, cancer cell–stroma crosstalk in the TME might be vital for many aspects of tumor aggression [69]. In addition, signaling via interactions between cancer cells and constituent cells in the TME might give rise to malignant properties [70]. In particular, fibroblasts recruited to the TME, known as cancer-associated fibroblasts (CAFs), are the largest components of the TME and have been well-studied with respect to cancer aggression [71]. Brechbuhl and colleagues recently showed that subtypes of CAFs exist in luminal breast cancer, and CD146-negative CAFs decrease the expression levels of estrogen receptor (ER) in ER-positive breast cancer cells and promote resistance to tamoxifen. In addition, the gene signature of breast cancer patients with CD146-negative CAFs correlates with poor prognosis in patients treated with tamoxifen [72]. More recently, an analysis of matrisome gene expression showed that CD146-negative CAFs are enriched with prometastatic proteins, including TNC [73]. Moreover, several previous studies have shown that high expression levels of TNC along with other CAF markers in the TME correlate with poor prognosis in several malignancies, such as prostate cancer [64], breast ductal carcinoma [61], and esophageal squamous cell carcinoma [58], indicating that TNC stimulates CAFs to promote cancer aggression. However, the substantial role of TNC in oncogenic transformation and malignant progression has not yet been clarified.

**Figure 1:**
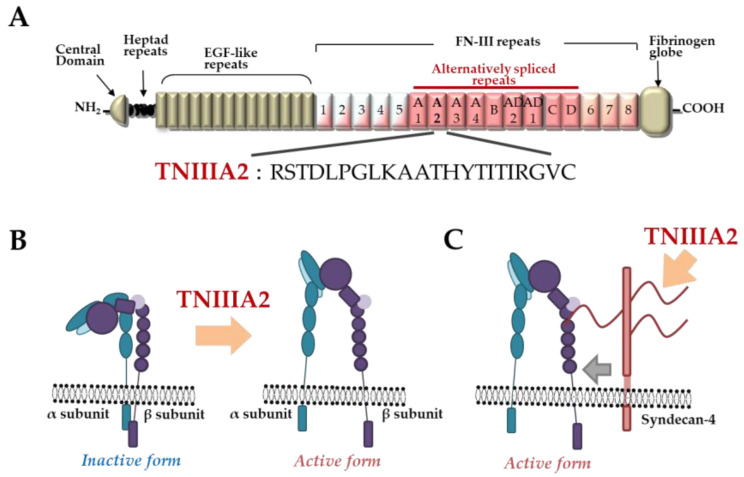
Tenascin-C (TNC)-derived TNIIIA2 fragments/peptides. (**A**) Schematic illustration of TNC and amino acid sequence of proadhesive peptide TNIIIA2. (**B**) Conformational shift of integrin activation by peptide TNIIIA2. (**C**) Lateral interaction of integrin with syndecan-4 ectodomain by peptide TNIIIA2.

**Figure 2:**
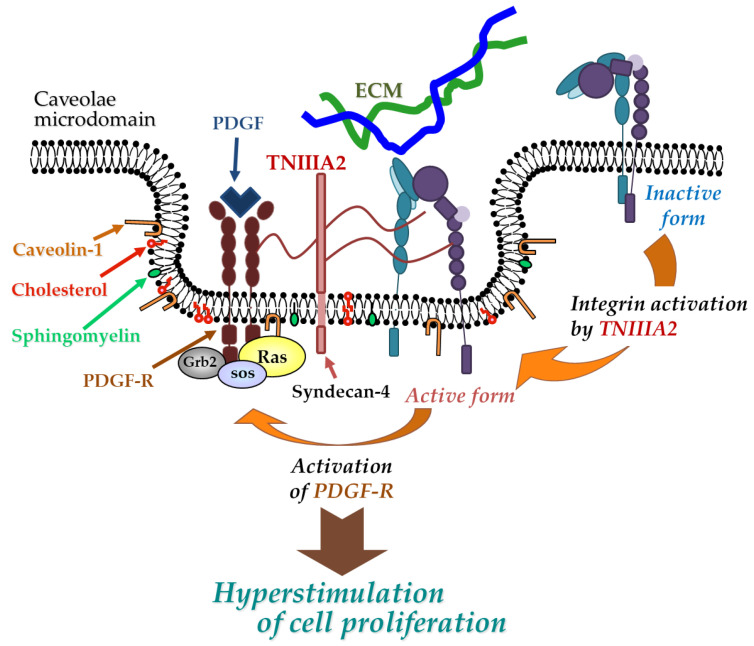
Physical and functional association between TNIIIA2-stimulated integrin α5β1 and PDGF receptor (PDGF-R). Peptide TNIIIA2 induces integrin α5β1 activation through a lateral association with syndecan-4, facilitating the formation of a molecular complex that includes activated integrin α5β1, syndecan-4, and activated PDGF-R in cholesterol- and caveolin-enriched membrane microdomains, which results in an enhanced activation of PDGF-R and leads to the hyperstimulation of cell proliferation.

TNC has been shown to have both proadhesive and antiadhesive properties in a context-dependent manner [49]. These underlying mechanisms remain elusive but could be explained in terms of the considerable diversity of TNC’s molecular forms. As shown in Figure 1A, TNC is composed of a central domain (assembly domain), epidermal growth factor (EGF)-like repeats, FN type III-like domains, and a fibrinogen globe-like domain, which are capable of interaction with ECM proteins, soluble factors, and cell receptors [54]. Moreover, human TNC contains 9 alternative splicing repeats in FN type III-like domains, and alternative splicing theoretically generates 511 possible splice variants [49], thus leading to the expression of TNC’s multifunctional activities in a context-dependent manner [54]. ECM remodeling often occurs in the TME and inflammatory regions where bioactive functions are released via cleavage by inflammatory proteinases [74]. TNC can also be proteolytically processed by matrix metalloproteinases (MMP) a disintegrin and metalloproteinase with thrombospondin motifs (ADAMTS), and alternative splicing repeats within FN type III repeats are particularly cleaved by MMP, which in turn exposes the specific bioactivity of TNC [49,74]. Among the TNC variants, those containing the FN type III repeats A2 are highly expressed in malignancies [75]. We previously found that FN type III repeats A2 of TNC molecules have cryptic sites composed of the amino acid sequence YTITIRGV (Figure 1A). Moreover, the 22-mer peptide TNIIIA2 containing its functional sites can induce the activation of integrin α5β1 through a lateral association with transmembrane heparan sulfate proteoglycan syndecan-4 (Figure 1B,C), which leads to the induction and potentiation of cell adhesion to the ECM [23]. This TNIIIA2-induced integrin activation is more potent and persistent than other known integrin activators [76]. Based on these effects, peptide TNIIIA2 was shown to influence various cellular functions. Notably, our results concerning the action of TNIIIA2 on survival and proliferation in stromal cells are interesting with regard to involvement in cancer progression. Peptide TNIIIA2 rendered NIH3T3 mouse nontransformed fibroblasts anoikis-resistant through integrin α5β1 activation-mediated prosurvival signaling [76]. Peptide TNIIIA2 also induced the platelet-derived growth factor (PDGF)-dependent dimerization of PDGF receptor (PDGF-R)β via the activation of integrin α5β1 to promote the PDGF-Rβ/Ras/mitogen-activated protein kinase (MAPK) signaling pathway, followed by the induction of hyperproliferation and the formation of dense multilayered cell aggregates—that is, transformed foci in NIH3T3 cells (Figure 2) [76]. These results suggest that TNIIIA2 has the ability to disrupt the normal cell phenotype. This raises the possibility that TNIIIA2-containing TNC fragments/peptides might be involved in oncogenic transformation and malignant progression.

### 2.1. Glioma/Glioblastoma

Glioblastoma multiforme (GBM) is the most common and aggressive primary glial tumor in adults. Despite multimodal therapies, including advanced surgery, radiotherapy, and chemotherapy, prognosis remains quite poor [77]. GBM is characterized by dysregulated proliferation and disseminative migration throughout the brain parenchyma, which hinders surgical resection. Thus, there is an urgent need for novel therapeutic strategies concurrent with the identification of underlying molecular mechanisms involved in aggressive progression.

As described above, several malignancies show high expression levels of TNC. Among them, GBM shows especially high levels [78]. In addition, TNC is expressed at significantly higher levels in mesenchymal GBM, which is the most aggressive phenotype of GBM [78]. It has been shown that TNC induces the enhanced proliferation of brain tumor-initiating cells [79], the promotion of migration in GBM cells [80,81], the modulation of angiogenesis in the GBM microenvironment [82], and the establishment of the immunosuppressive microenvironment of GBM through the inhibition of T cell activity [78,83], which is involved in GBM aggressiveness. In fact, TNC expression correlates to poor prognosis in GBM, and its expression is considered a poor prognostic factor. Moreover, a number of previous studies have found that PDGF and PDGF-R are involved in GBM aggression [84,85]: PDGF and PDGF-R are implicated in the self-renewal and tumorigenicity of GBM in an autocrine/paracrine manner [86,87,88]. In addition, an analysis of clinical samples showed that some GBM subgroups showed high levels of PDGF-B, which is a phosphorylated form of PDGF-Rβ [89]. PDGF-Rβ is highly expressed at the invasive tumor front in GBM cells with acquired resistance to antiangiogenic therapy [90]. However, the substantial functions of TNC in GBM aggression have not been established, and few studies have focused on the relationship between TNC and PDGF signaling in GBM aggression.

**Figure 3:**
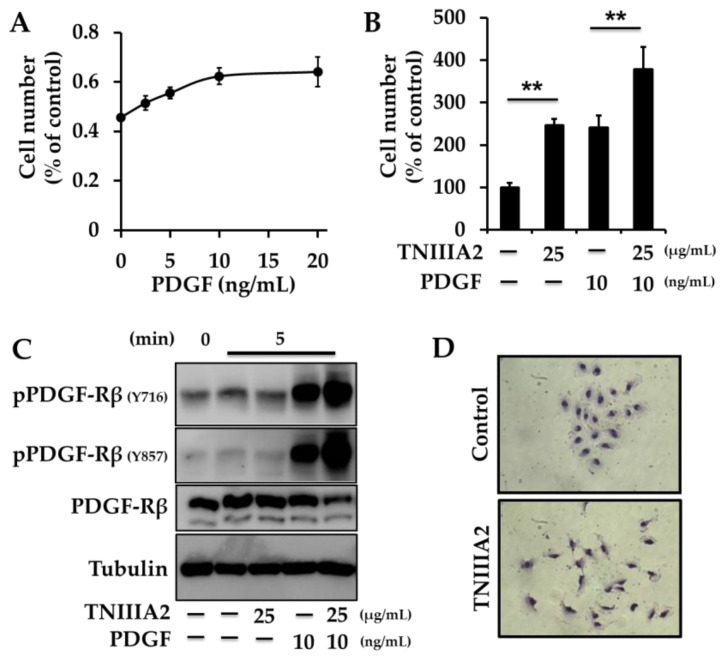
Peptide TNIIIA2 induces the hyperproliferation and disseminative migration of glioma/glioblastoma multiforme (GBM) cells. (**A**,**B**) Effect of peptide TNIIIA2 on PDGF-dependent proliferation of glioma cells. Rat glioma C6 cells were stimulated with PDGF in the presence or absence of peptide TNIIIA2 for 2 days. Cells were subjected to WST-8 assay. Each point represents the mean ± SD, ** *p* < 0.01. (**C**) C6 cells on fibronectin substrate were stimulated with peptide TNIIIA2, PDGF, or their combination, for the indicated period. Cell lysates were subjected to Western blotting analysis. (**D**) Scattering assay was performed. Cobblestone-like cell clusters were developed by culturing human GBM U251 cells on fibronectin substrate. Cells were treated in the presence or absence of TNIIIA2 (25 μg/mL) for 12 h.

More recently, we found that a peptide containing the bioactive site of TNC, TNIIIA2, can potently activate β1-integrin in GBM cells. Based on this effect, TNIIIA2 renders GBM cells with the properties of dysregulated proliferation: PDGF stimulated cell proliferation in a concentration-dependent manner but reached a plateau at about 10–20 ng/mL (rat glioma C6 cells: Figure 3A, human GBM T98G cells, and rat glioma 9L cells: Ref. [91]). The PDGF-stimulated proliferation at submaximal concentration was further enhanced by the addition of TNIIIA2 (rat glioma C6 cells: Figure 3B, human GBM T98G cells and rat glioma 9L cells: Ref. [91]). TNIIIA2-stimulated PDGF-dependent cell proliferation was specifically abrogated by functional-blocking antibodies against integrin α5 and β1 subunits, but not against integrin αv or β3 subunits. Likewise, similar to the action of TNIIIA2, β1-integrin-activating antibody also promoted the PDGF-dependent hyperproliferation, indicating that GBM cell proliferation secured by PDGF stimulation is heavily promoted by TNIIIA2 via integrin α5β1 activation. Mechanistically, immunoprecipitation and confocal microscopy analyses showed that integrin α5β1 activated by TNIIIA2 both physically and functionally cooperated with PDGF-stimulated PDGF-Rβ and, consequently, PDGF-Rβ was hyperactivated, which led to the stimulation of Ras-MAPK and Akt signaling pathways (rat glioma C6 cells: Figure 3C, human GBM T98G cells and rat glioma 9L cells: Ref. [91]). Furthermore, anchorage-independent growth is thought to be a malignant property of cancer cells. TNIIIA2-stimulated PDGF-dependent hyperproliferation was further substantiated in anchorage-independent cell growth. Collectively, peptide TNIIIA2 seems to be capable of maximizing growth factor signaling, leading to the dysregulated proliferation of GBM cells.

TNIIIA2 also promoted the proliferation only of GBM cells expressing PDGF-Rβ, even without the addition of exogenous PDGF. Mechanistically, TNIIIA2 induced the upregulation of PDGF levels, which in turn stimulated the upregulation of TNC, which is the parental molecule of TNIIIA2. Moreover, induced TNC upregulated the expression of MMP, which has the ability to liberate TNIIIA2 from the TNC molecule. Thus, the TNC–MMP–TNIIIA2–PDGF positive spiral loop may function in GBM, and thus contribute to dysregulated proliferation, which is one of the hallmarks of GBM cells [92].

The aggressive phenotype of GBM is also characterized by disseminative migration. TNIIIA2 induced disseminative migration, as determined by wound healing assay (human GBM T98G cells and rat glioma 9L cells: Ref. [91], human GBM U251 cells, rat glioma C6 cells and mouse glioma cells GL261 cells: Ref. [92]) and cell scattering assay (human GBM U251 cells: Figure 3D, human GBM T98G cells and rat glioma 9L cells: Ref. [91]). This TNIIIA2-induced disseminative migration was abrogated by function-blocking antibodies against β1-integrin, or RGD peptide, which is an antagonist of integrin α5β1 [91]. Taken together, one of the mechanisms underlying the TNC-induced disseminative migration of GBM cells might be attributed to the β1-integrin activation triggered by TNIIIA2-containing TNC fragments/peptides.

### 2.2. Colitis-Associated Colorectal Cancer

It has been established that patients with inflammatory bowel disease (IBD), including ulcerative colitis (UC) and Crohn’ disease, have an increasing risk of developing colitis-associated colorectal cancer (CAC) [93]. Unlike sporadic colorectal cancer, which involves an adenoma–carcinoma sequence, the molecular basis for the onset of CAC remains unclear [94]. It has been shown that patients with CAC exhibit a poorer outcome than patients with sporadic colorectal cancer [95]. Thus, there is an urgent need for novel therapeutic and prophylaxis strategies concurrent with the identification of underlying molecular mechanisms involved in the onset of CAC. TNC expression levels are reported to be elevated both in areas of ulceration in UC and in areas of stricture in Crohn’s disease [96]. Moreover, TNC was strongly expressed in the region of colitis with dysplasia in a mouse model of azoxymethane (AOM)–dextran sulfate sodium (DSS)-induced CAC [97]. Therefore, it is conceivable that TNC may contribute to the pathogenesis of CAC. We addressed the pathological relevance of TNIIIA2 related-functional fragments/peptides for the onset of CAC [98]. In the AOM-DSS mouse model, the expression of the TNIIIA2-containing TNC fragments/peptides was detected in dysplastic lesions in the mucosal stroma, speculating that the stimulation of TNIIIA2-containing TNC fragments/peptides might influence the preneoplastic development of lesions in CAC. Therefore, we focused on the effect of TNIIIA2 on both preneoplastic epithelial cells and stromal fibroblasts in in vitro experiments. Interestingly, while TNIIIA2 did not show a significant direct effect on preneoplastic cells, TNIIIA2-stimulated fibroblasts secreted a paracrine factor(s), leading to the promotion of survival/proliferation in preneoplastic cells, as determined by a 2D co-culture system and conditioned medium experiments. Similar phenomena of the effect of TNIIIA2-stimulated fibroblasts on growth in preneoplastic cells were observed for colon cancer cell lines. Taken together, although further investigations will be needed to identify the paracrine factor(s) secreted by TNIIIA2-stimulated fibroblasts, TNIIIA2-containing TNC fragments/peptides might be implicated in the development of CAC [98].

## 3. Fibronectin-Derived Peptide, FNIII14

**Figure 4:**
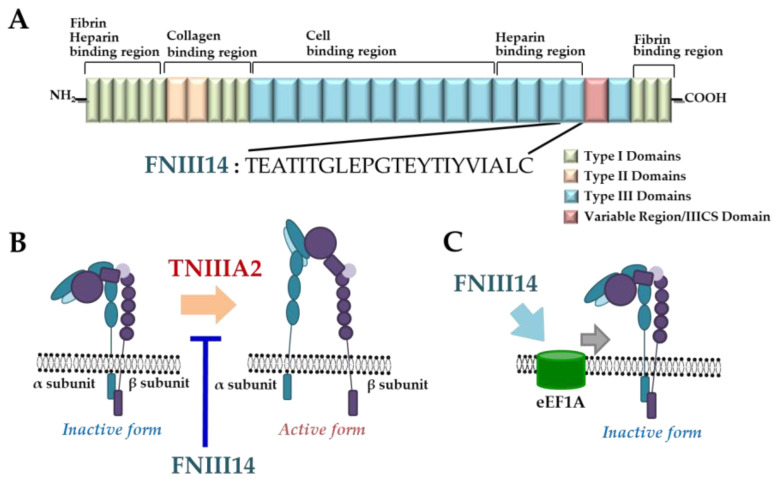
Plasma fibronectin-derived peptide FNIII14. (**A**) Schematic illustration of plasma fibronectin and amino acid sequence of antiadhesive peptide FNIII14. (**B**) Conformational shift of integrin by peptide FNIII14. (**C**) Eukaryotic elongation factor 1A (eEF1A) as a putative membrane receptor of peptide FNIII14.

FN is one of the most abundant and ubiquitous ECM proteins [99]. In particular, pFN is regarded as a matricellular protein because it can regulate cell functions via bioactive fragments within its molecules as well as function as a humoral factor involved in hemostasis and thrombosis. With regard to the functional sites within FN, the sequence Leu–Asp–Val (LDV) in the CS-1 region of type IIICS connecting-segment domain and the sequence RGD in the 10th type III repeat, which are recognized by integrin α4β1 and α5β1, respectively, have been well-characterized [99]. Besides these, there are observations of several bioactive sequences and functions (Table 1) [100]. As shown in Figure 4A, FN contains fibrin-, heparin-, collagen-, and cell-binding regions, each of which comprises type I, II, and III domains. We previously also found that pFN harbors a cryptic functional site, termed FNIII14, corresponding to the amino acid sequence YTIYVIAL within the 14th FN type III domains (Figure 4A) [48]. A 22-mer FN peptide containing site FNIII14 has the ability to change β1-integrin conformation from the active to the inactive form to induce functional inactivation in an entirely opposite manner to TNIIIA2 (Figure 4B) [101]. Based on these effects, peptide FNIII14 was shown to influence various cellular behaviors via the inactivation of β1-integrin [91,98,101]. Surprisingly, we previously found that a minor part of eukaryotic translation elongation factor 1A (eEF1A) is localized on the cell surface and acts as a membrane receptor for FNIII14, that is, the association of β1-integrin with the cell surface of eEF1A in response to FNIII14 induces the functional inactivation of β1-integrin (Figure 4C) [17]. It has been well established that eEF1A plays a critical role during protein biosynthesis on ribosomes [102]. However, besides this canonical role, our findings suggest that non-canonical eEF1A functions also contribute to cell regulation as membrane receptors, thereby affecting various cellular behaviors.

The effects of pFN-derived peptide FNIII14 on cell regulation, especially its antitumor applications via the inactivation of integrins, have been reported (Table 2), and these reports have suggested some of the implications for novel therapeutic approaches targeting β1-integrin activation.

### 3.1. Glioma/Glioblastoma

**Figure 5:**
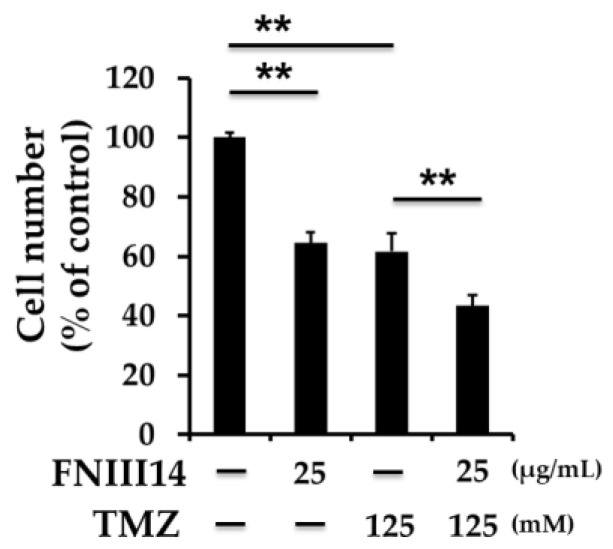
Peptide FNIII14 augments a TMZ-induced antitumor effect. Mouse glioma GL261 cells were treated with the indicated concentrations of peptide FNIII14 in the presence or absence of TMZ for 3 days. Cells were subjected to WST-8 assay. Each point represents the mean ± SD, ** *p* < 0.01.

Our findings showed that FNIII14 can induce the conformational changes necessary in β1-integrins for functional inactivation in GBM cells. Moreover, FNIII14 abolished the proadhesive effects of TNC-derived peptide TNIIIA2 by inducing the inactivation of β1-integrins. Based on these effects, FNIII14 impeded the acquired malignant properties through β1-integrin activation by TNIIIA2, such as hyperproliferation and disseminative migration, which are features of GBM aggression [91]. Furthermore, FNIII14 monotreatment delayed tumor growth in a rat glioma 9L cells subcutaneous mouse xenograft model. Moreover, we found that FNIII14 sensitizes GBM cells to the DNA alkylating agent temozolomide (TMZ), which is the first-line chemotherapeutic agent for GBM therapy (mouse glioma GL261 cells: Figure 5, T98G and 9L cells: Ref. [91]). It is known that resistance to TMZ is due, at least in part, to the enhancement of DNA repair by O^6^-methylguanine-DNA methyltransferase (MGMT) [91]. FNIII14 is capable of inducing the downregulation of MGMT at the mRNA and protein levels in a MGMT promoter methylation-independent manner, which contributes to TMZ sensitization. In addition, it also augments TMZ-induced cytotoxicity in the rat glioma 9L cells subcutaneous mouse xenograft model. It has been reported that the expression levels of α5-integrin increase with glioma grade [108,109,110,111] and correlate with poor prognosis in high-grade glioma/GBM [108]. In particular, integrin α5β1 is expressed at significantly higher levels in mesenchymal GBM, which is the most aggressive subtype of GBM [110]. It has also been shown that the activation of α5-integrin induces cell dissemination [112], which is an antagonist for integrin α5β1 or depletion of α5-integrin sensitizes GBM cells to TMZ via modulating the p53 pathway [108,113], and that β1-integrin inhibition potentiates antiangiogenic therapy [114]. Taken together with these observations and our results, the application of FNIII14 targeting integrin α5β1 as a monotherapy or in combination regimens could represent promising therapeutic strategies for GBM therapy.

### 3.2. Colitis-Associated Colorectal Cancer

As described above, TNIIIA2 stimulated fibroblasts to enhance the survival/proliferation of preneoplastic epithelial cells in in vitro experiments. FNIII14 induced the inactivation of β1-integrin in fibroblasts and inhibited the effects of TNIIIA2-stimulated fibroblasts on the enhancement of proliferation in preneoplastic cells. We also recently found that FNIII14, which inactivates β1-integrin, can suppress the development of polyps in an AOM-DSS mouse model [98], perhaps through inhibition of the function of stromal fibroblasts [115]. It has recently been reported that the administration of ATN-161, an antagonist that binds integrin α5β1 and αvβ3, suppressed tumorigenesis in CAC through the inhibition of angiogenesis in the AOM-DSS mouse model [97], and that fucoxanthin, which is a carotenoid with strong antitumor activity, induces anoikis to suppress the incidence and multiplicity of colonic adenocarcinoma in the AOM-DSS mouse model through the attenuation of β1-integrin signaling [116]. This indicates the importance of β1-integrin signaling in CAC therapy. Taken together, FNIII14 may be a promising agent for the prophylaxis and therapeutic treatment of CAC.

## 4. Perspectives and Future Directions

Several experimental results led us to speculate that exposure to TNIIIA2-containing TNC fragments/peptides is involved in cancer aggression. The amounts of large variant TNCs or the extent of TNC degradation have been associated with poorer prognosis in patients with malignancies [117,118,119]. In addition, many studies have demonstrated that ECM stiffening enhances integrin signaling, which leads to the malignant progression of tumor cells [120,121]. Increasing evidence on the correlation between tumor stiffness and malignant aggression will provide important insights into the substantial functions of TNC. An insightful study on the role of TNC in ECM stiffness in the GBM microenvironment has been previously published [122]. Barnes and colleagues recently found that GBM has an increased bulky glycocalyx, TNC-enriched stiffened ECM, and promoted integrin signaling [122]. They further found that tumor xenografts derived from GBM cells expressing an auto-clustered active mutant β1-integrin (V737N) show enhanced integrin mechanosignaling, promoted TNC-enriched ECM stiffness, and led to increased tumor burden [122]. These consequences might be explained by the effect of TNIIIA2-containing TNC fragments/peptides on β1-integrin activation, because TNIIIA2 seems to be able to induce the clustering of β1-integrin on cell membranes [23]. It is unlikely that at least the antiadhesive activity of TNC, which has been considered a major bioactive function of this molecule, is responsible for the increased ECM stiffness and consequent enhanced integrin signaling. Further investigations will be needed to determine whether TNIIIA2-induced integrin activation actually contributes to increased ECM stiffness.

TME consists of ECM molecules, cancer cells, and various other cells such as CAFs, immune cells, and vascular cells. Given that CAFs are the major components of the TME, and that intercellular communication between cancer cells and CAFs is critically involved in cancer progression, the crosstalk between cancer cells and CAFs is currently the focus of intensive study. Targeting the molecular mechanisms that activate CAFs may represent efficient anticancer strategies [123]. Our findings suggest that TNC-derived TNIIIA2 shows direct and indirect responses to the malignant progression of cancer cells: TNIIIA2 acts not only directly on cancer cells to enhance cancer progression, but also on fibroblasts, and its secretome subsequently influences the malignant properties of cancer cells. Hence, the inhibition of TNIIIA2 activity might result in an effective induction of anticancer properties. Therefore, function-blocking antibody against TNIIIA2 [76] or FNIII14 would be a promising strategy for cancer therapy.

To date, many synthetic peptides based on FN bioactive sequences, which are mostly derived from the RGD sequence within the 10th FN type III domains or PHSRN sequence within the 9th FN type III domains, have been widely attempted in clinical studies as antitumor agents [109]. Cilengitide (EMD 121974), a cyclic RGD pentapeptide, is a selective integrin antagonist for αvβ3 and αvβ5, and acts in an antiangiogenic manner [109]. Cilengitide was evaluated in clinical trials in patients with GBM [124,125,126,127,128], head and neck squamous cell carcinoma [129,130], non-small-cell lung carcinoma [131], and prostate cancer [132,133]. In addition, ATN-161 (Ac-PHSCN-NH_2_) derived from the synergy region of fibronectin is a selective antagonist for integrin α5β1 and is antiangiogenic and antimetastatic [29,134,135,136]. ATN-161 was evaluated in a phase I/II trial for recurrent malignant glioma (ClinicalTrials.gov Identifier: NCT00352313) and a phase II trial for advanced renal cell cancer (ClinicalTrials.gov Identifier: NCT00131651). Despite great efforts made in preclinical studies, numerous clinical trials have unfortunately failed to demonstrate significant therapeutic benefits [110]. These FN-based antagonists generally have the ability to enact competitive interference with the binding of integrin αvβ3/αvβ5 or α5β1 to ECM proteins. Based on these effects, the antagonists have been shown to promote antiangiogenic activity and ECM detachment-induced apoptosis, which is called anoikis. However, malignantly transformed cells often acquire the nature of anchorage-independent growth, namely, anoikis resistance in the TME [137]. Hence, it is conceivable that integrin competitive antagonists may not efficiently induce cell death in anoikis-resistant cells. In fact, our previous study showed that TNIIIA2 renders cancer cells resistant to apoptosis under cell-detached conditions, and FNIII14 could inhibit TNIIIA2-induced resistance to apoptosis, although the RGD peptide did not show such an effect. Unlike the RGD peptide, FNIII14 is capable of inducing conformational change in β1-integrin from the active to inactive state, consequently impairing integrin-mediated survival signaling in both attached and suspended cells [91]. Although the possibilities of integrin competitive antagonists need to be pursued further, FNIII14 could have the potential to overcome these issues of integrin competitive antagonists via the conformational inactivation of β1-integrin.

To further determine whether FNIII14 has potential clinical applications, we are attempting to develop modifications to improve its absorption, distribution, metabolism, and excretion (ADME) and pharmacokinetic properties. Synthetic peptides are susceptible to degradation by serum peptidases, such as carboxypeptidases and endopeptidases in blood plasma. In order to make the peptides resistant to these peptidases and to improve stability in human blood plasma, the N-terminal E (Glu) amino acid residue of FNIII14 (TEATITGLEPGTEYTIYVIAL) was replaced with it non-natural D-form, which is not recognized by common peptidases. This modification resulted in the improved stability of the peptide in human blood plasma. Moreover, in terms of safety, FNIII14-treated mouse (1 mg intravenous route) did not exhibit myelotoxicity [104]. In addition, in several of our mouse models, body weight did not change in response to dosing with FNIII14 [91,98,103]. Further evaluations involving, for example, pharmacokinetic/pharmacodynamic (PK/PD) analyses and non-clinical toxicity studies examining of genotoxicity and carcinogenicity are needed.

## 5. Conclusions

ECM molecules are released as bioactive fragments through proteolytic cleavage by inflammatory proteinases in the context of the TME. The peptides containing these bioactive fragments show unique biological activities, which are often not detected in the parent molecules. Therefore, these bioactive peptides are employed in research studies into the mechanisms of cancer aggression and the development of cancer therapeutics. TNC is highly expressed in the TME and the peptide containing the bioactive site of TNC, TNIIIA2, contributes to cancer aggression through β1-integrin activation both potently and persistently. Moreover, unlike integrin competitive antagonists, the peptide containing the bioactive site of pFN, FNIII14, can induce a conformational change in β1-integrin from the active to the inactive state, thus contributing to the disruption of cancer aggression. Peptide FNIII14 could be a promising therapeutic approach.

## Figures and Tables

**Table 1:** Tenascin-C or fibronectin-derived bioactive sequences.

Parental Molecule	Sequence	Function (In Vitro and In Vivo Settings)	Ref.
***Tenascin-C***	VFDNFVLK	Neurite outgrowth	[18]
	VSWRAPTA	Glioma cell migration, neuronal branching	[19]
	PLAEIDGIELTY	Cell adhesion, binding to integrin α9β1	[20]
	VSGNTVEYALPTLE	Fibroblast proliferation	[21]
	LDSPTAPTVQSTALTWRP	Fibroblast and endothelial cell proliferation	[21]
	WYRNCHRVNLMGRYGDNNHSQGVNWFHWKG	Cell adhesion, binding to integrin αvβ3	[22]
	RSTDLPGLKAATHYTITIRGVC (TNIIIA2)	Cell adhesion, integrin activation Enhancement of lung metastatic nodule formation in a mouse model of metastasis of colon cancer cells	[23] [24]
***Fibronectin***	RGD	Cell adhesion Antiangiogenic effect in vivo	[25] [26]
	LDV	Cell adhesion, binding to integrin α4β1	[27]
	PHSRN	Synergistic interactions between integrin α5β1 and RGD Antiangiogenic effect in vivo	[28] [29]
	REDV	Binding to integrin α4β1	[30]
	SLLISWD	Fibronectin fibril assembly	[31]
	KLDAPT	Binding to integrin α4β1 and α4β7	[32]
	EDGIHEL	Binding to integrin α4β1 and α9β1	[33]
	IDAPS	Binding to integrin α4β1	[34]
	ALNGR	Cell adhesion, binding to β1-integrn	[35]
	WQPPRARI	Cell adhesion, binding to heparin	[36]
	SRNRCNDQ	Plasminogen activation	[37]
	KNEED	Cell adhesion, cell-recognition site	[38]
	RWRPKNSVGR	Cell spreading, cell growth, vasodilation	[39]
	PSHISKYILRWRPK	Binding to PDGF-BB, cell survival	[40]
	YEKPGSPPREVVPRPRPGV	Cell adhesion, heparin-binding region	[41]
	KNNQKSEPLIGRKKT	Heparin-binding region, neurite outgrowth	[41]
	YRVRVTPKEKTGPMKE	Cell adhesion, heparin-binding region	[41]
	AHEEICTTNEGVM	Matrix assembly, cell migration	[42]
	ETTIVITWTPAPR	Cell adhesion, binding to MIA protein Reduction of the size of lung nodules in a mouse model of melanoma metastasis	[43] [44]
	TSLLISWDAPAVT	Cell adhesion, binding to MIA protein	[43]
	NSLLVSWQPPRAR	Cell adhesion, binding to MIA protein	[43]
	GTQSTAIPAPTD	Cell adhesion, binding to MIA protein Reduction of the size of lung nodules in a mouse model of melanoma metastasis	[44]
	PRARIY	Cell adhesion, neuroprotective effect	[45]
	NVSPPRRARVTDATETTITISW	Binding to heparin	[46]
	VTEATITGLEPGTEYTIY	Binding to DPPIV Reduction of lung colonization in a mouse model of metastasis	[47]
	TEATITGLEPGTEYTIYVIAL (FNIII14)	Cell adhesion, integrin inactivation Antitumor effects in vivo (Table 2)	[48]

PDGF, platelet-derived growth factor; MIA, melanoma inhibitory activity; DPP, dipeptidyl peptidase.

**Table 2:** Antitumor effects of peptide FNIII14 under in vitro and in vivo settings.

Cancer Type	Cell Type/Animal Model	Phenotypic Effects	Ref.
***Glioma***	T98G, 9L cells	Suppression of cell survival/proliferation	[91,92]
***/Glioblastoma***	T98G	Suppression of disseminative migration	[91]
	T98G, 9L cells	Potentiation of temozolomide (TMZ) cytotoxicity	[91]
	T98G cells	Downregulation of O^6^–methylguanine–DNA methyltransferase (MGMT) levels	[91]
	Mouse subcutaneous xenograft (9L cells)	Suppression of tumor growth as monotherapy	[91]
	Mouse subcutaneous xenograft (9L cells)	Potentiation of TMZ action	[91]
***Neuroblastoma***	IMR-32, NB-1, KELLY cells	Downregulation of N-myc levels by proteasomal degradation	[103]
	IMR-32 cells	Suppression of cell survival/proliferation	[103]
	Mouse subcutaneous xenograft (IMR-32 cells)	Suppression of tumor growth as monotherapy	[103]
***Colitis-associated colorectal cancer (CAC)***	Azoxymethane–dextran sodium sulfate (AOM-DSS) mouse model	Suppression of polyp development as monotherapy	[98]
***Acute myelogenous leukemia (AML)***	U937, HL-60, Fresh leukemic cells from AML patients	Disruption of cell adhesion-mediated drug resistance (CAM-DR) to cytosine arabinoside (Ara C)	[104]
	Mouse model of minimal residual disease (MRD) in AML (U937 cells)	Eradication of bone marrow MRD in mice transplanted with U937 cells and improvement of survival mouse treated with Ara C	[104]
***Lymphoma***	L5178Y-ML25 cells	Inhibition of cell migration	[105]
	Mouse model of experimental tumor metastasis (L5178Y-ML25 cells)	Inhibition of the liver and spleen metastases as monotherapy	[105]
***Mammary tumor***	4T1 cells	Potentiation of doxorubicin (Dox) cytotoxicity	[106]
	Mouse model of experimental tumor metastasis (4T1 cells)	Inhibition of the liver metastases when coadministered with Dox	[106]
***Melanoma***	B16BL6 cells	Increasing chemosensitivity of antitumor drugs (e.g., Aclarubicin, Vinblastine, 5-Fluorouracil (5-FU))	[106]
***Oral squamous cell carcinoma (OSCC)***	Ca9-22/FR2 cells	Potentiation of 5-FU cytotoxicity	[107]

T98G, human GBM cell line; 9L, rat gliosarcoma cell line; C6, rat glioma cell line; U251, human GBM cell line; GL261, mouse glioma cell line; IMR-32, human neuroblastoma cell line; NB-1, human neuroblastoma cell line; KELLY, human neuroblastoma cell line; U937, human acute myelocytic leukemia cell line; HL-60, human acute myelocytic leukemia cell line; L5178Y-ML25, murine T lymphoma cells; 4T1, mouse mammary tumor cell line; B16BL6, mouse melanoma cell line; Ca9-22/FR2, 5-FU-resistant OSCC cell line.

## Abbreviations

ADAMTS	a disintegrin and metalloproteinase with thrombospondin motifs
ADME	absorption, distribution, metabolism and excretion
AML	acute myelogenous leukemia
AOM	azoxymethane
Ara C	cytosine arabinoside
CAC	colitis-associated cancer
CAFs	cancer-associated fibroblasts
CAM-DR	cell adhesion-mediated drug resistance
Dox	doxorubicin
DPPIV	dipeptidyl peptidase IV
DSS	dextran sulfate sodium
ECM	extracellular matrix
eEF	eukaryotic elongation factor
EGF	epidermal growth factor
ER	estrogen receptor
FN	fibronectin
GBM	glioblastoma multiforme
IBD	inflammatory bowel disease
LDV	Leu–Asp–Val
MAPK	mitogen-activated protein kinase
MCAM	melanoma cellular adhesion molecule
MGMT	O6-methylguanine–DNA methyltransferase
MIA	melanoma inhibitory activity
MMP	matrix metalloproteinase
MRD	minimal residual disease
OSCC	oral squamous cell carcinoma
PDGF	platelet-derived growth factor
PDGF-R	platelet-derived growth factor-receptor
pFN	plasma fibronectin
PD	pharmacodynamics
PK	pharmacokinetics
RGD	Arg–Gly–Asp
SPARC	secreted protein acidic and rich in cysteine
TLR	toll-like receptor
TME	tumor microenvironment
TMZ	temozolomide
TNC	tenascin-C
UC	ulcerative colitis
5-FU	5-Fluorouracil
